# Improving access to innovation from international health technology assessment

**DOI:** 10.1017/S0266462325100135

**Published:** 2025-06-02

**Authors:** Carlos Crespo

**Affiliations:** 1 Department of GM Statistics, University of Barcelona, Barcelona, Spain; 2 Axentiva Solutions, Barcelona, Spain

**Keywords:** Value assessment, early HTA, Efficiency, Budget Impact

## Abstract

The field of health care has evolved from an emphasis on evidence-based medicine, with a focus on efficacy, safety, and tolerability, to the pursuit of evidence-based efficiency and sustainable innovation in many respects (healthcare budgets, carbon print, etc.). This evolution can be attributed, in part, to the contributions of health technology assessment (HTA) bodies, which have facilitated the incorporation of various factors into the decision-making process (1). These factors include comparative effectiveness, quality of life, efficiency, budgetary impact, and organizational impact, among others. Within the domain of health care, irrespective of the perspective of each entity (e.g., Food and Drug Administration (FDA), European Medicines Agency, etc.), there is an imperative for the presence of evidence and its assessment in the most transparent manner possible, with the objective of ensuring the incorporation of healthcare technologies.

The field of health care has evolved from an emphasis on evidence-based medicine, with a focus on efficacy, safety, and tolerability, to the pursuit of evidence-based efficiency and sustainable innovation in many respects (healthcare budgets, carbon print, etc.). This evolution can be attributed, in part, to the contributions of health technology assessment (HTA) bodies, which have facilitated the incorporation of various factors into the decision-making process (1). These factors include comparative effectiveness, quality of life, efficiency, budgetary impact, and organizational impact, among others. Within the domain of health care, irrespective of the perspective of each entity (e.g., Food and Drug Administration (FDA), European Medicines Agency, etc.), there is an imperative for the presence of evidence and its assessment in the most transparent manner possible, with the objective of ensuring the incorporation of healthcare technologies.

This has led to the conclusion that to promote innovation in health, as a tool to improve health systems and the population’s well-being, it is necessary to encourage early dialogue among the different stakeholders in the sector in an effort to optimize, accelerate, and maximize the benefits of health technologies. Ensuring access to the most effective health technologies for the appropriate patients in the most efficient manner for the health system, while taking into consideration the technical and operational capacity of the health system, is also fundamental.

The HTA group (2) has highlighted the need to establish a common framework defining what early HTA is, as a first step to provide a common anchor for researchers and developers to optimize their resources and be of benefit to society at large. Unlike other assessments, this is a process rather than a final milestone. We have to take into account that there are several phases of preconcept, prototyping, clinical development, and pharmacoeconomic evidence before the technology is on the market, leading to the first version with minimum value (minimum viable technology), which can be improved by incremental innovation once it is on the market. For this reason, whether for the need to improve development; evidence; or to obtain funding (from angel investors, venture capital, investment funds, etc.), early HTA is a process that should help researchers shape their value proposition for society. It is not about generating value in a spurious way, as we are see in some cases with artificial intelligence and other technologies (3), which are sometimes based more on magnifying the benefit from advertising arguments than on duly justified necessity, but to ensuring that, in the development of health technologies, clinical and nonclinical aspects have been evaluated with the highest possible degree of evidence, to avoid surprises in HTA evaluations or, in the case of Europe, in the Joint Clinical Assessment (4).

Value assessment from an HTA point of view is under constant review. Long after the first definitions of HTA assessment and the publication of Drummond’s book on Economic Evaluation of Health Care Programs (5;6), different definitions of value have appeared at the macro level, such as the one proposed by Potter (7), to the present day, where International Society for Pharmacoeconomics and Outcome Research has proposed a flower of the value (8) with petals that are even linked to value of hope and other variants that try to emphasize the social perspective (9;10). They are all aimed at the provision of health services and the uptake of health technologies, but they do not have such a clear focus on development through risk mitigation and optimizing market access as early HTA. To bridge this gap between the development process and final evaluation, many organizations have promoted initiatives or programs to assist researchers. In this regard, the FDA has the Breakthrough Therapy Designation and Breakthrough Device Program, a program that helps identify unmet needs by guiding development pathways; the National Institute for Health and Care Excellence has the Early Dialogue within its Scientific Advice Program, which includes the generation of evidence aligned with the requirements of HTA. Others have addressed the importance of improving integration and cooperation between three key processes in health care: regulation, HTA, and the development of clinical guidelines (11). Although these processes are independent, they share a common evidence base, and their alignment can be of great help to developers.

One of the keys to early HTA, and this is emphasized in the manuscript (2), is that this process attempts to identify the essential elements where the evidence needs to be improved and to identify the key parameters that will be amenable to the final decision-making. Although the economic evaluation at this stage is not based on evidence but on potential scenarios, it is a very useful exercise for the developer that allows him or her to focus on the development of his or her product. In the end, all aspects of early HTA, whether clinical, economic, or other aspects linked to unmet needs among others, will help developers to understand the value of their product not only for themselves but also for society and potential investors – key to providing value for money and rapid access to patients.

Within early HTA, health technology must be evaluated in each of the Magnitude, budget Impact, Relevance, and Efficiency (MIRE) attributes to successfully demonstrate value.

**M**agnitude: The target therapeutic market is a critical consideration in the early stages of health technology development. It involves assessing the current and potential market in line with potential competitors and unmet needs.

Budget **I**mpact: Financial modeling is a valuable tool in the initial stages of health therapy development, as it enables companies to simulate the potential market and the impact of the health technology on it. In addition, it facilitates the identification of the return on investment.

**R**elevance: Understanding the burden of disease is essential as it allows developers not only to assess the impact of the disease on patients and society but also to evaluate the clinical impact that the new technology may have.

**E**fficiency: The cost-effectiveness of a new technology is a critical consideration in its development, as it facilitates the identification of the potential market price and key parameters.

Although terminology has been subject to debate, as exemplified by the difficulty of reaching a consensus on a shared definition, its use is very useful. Employment may serve to heighten awareness among developers and further cultivate collaboration between institutions, as well as public–private collaboration. Moreover, the term’s usage in publications will facilitate the identification of use cases that may align with developers’ needs.

There is a need to bring together the efforts of all those involved because, in addition to improving the health of society, investment in health technologies can generate improvements in economic growth, can even generate long-term savings, and can be a focus for improving the equity of our healthcare systems. Given the different incentives available to investors, we must all be able to promote investment in health technologies because of their great added value. It is essential to acknowledge that investment in health technologies is not merely a financial expenditure; rather, it constitutes a strategic allocation of resources with the potential to generate substantial returns across diverse societal sectors and to make the system more robust/resilient to unforeseen events because it streamlines according to relevance and generates a clear and traceable path. A healthier society is a more equitable and wealthier society.

## References

[1] Henshall C, Schuller T. Health technology assessment, value-based decision making, and innovation. Int J Technol Assess Health Care. 2013;29:353–359. 10.1017/S0266462313000378 23845404

[2] Grutters JPC, Bouttell J, Abrishami P, et al. Defining early health technology assessment: Building consensus using delphi technique. Int J Technol Assess Health Care. 2025;1–27. 10.1017/S0266462325100123 PMC12178750 40452233

[3] Matheny M, Israni ST, Ahmed M, Whicher D, editors. Artificial intelligence in health care. Washington, D.C.: National Academies Press; 2019. 39146448

[4] Schuster V. EU HTA regulation and joint clinical assessment—Threat or opportunity? J Mark Access Heal Policy. 2024;12:100–104. 10.3390/jmahp12020008 PMC11130920 38808311

[5] Drummond MF, Stoddard GL, Torrance GW. Methods for the economic evaluation of health care programmes. Oxford, United Kingdom: Oxford University Press. 3rd ed. 1988.

[6] Jonsson M, Alban A. *Methods for the Economic Evaluation of Health Care Programmes*, M. F. Drummond, G. L. Stoddard, and G. W. Torrance. Oxford: Oxford University Press, 1987, 182 pp., $29.50. Int J Technol Assess Health Care. 1988:4. 10.1017/s026646230000773x

[7] Davidson A, Randall RM. Michael Porter and Elizabeth Teisberg on redefining value in health care: An interview. Strateg Leadersh. 2006;34:48–50. 10.1108/10878570610711288

[8] Lakdawalla DN, Doshi JA, Garrison LP, Phelps CE, Basu A, Danzon PM. Defining elements of value in health care—A health economics approach: An ISPOR special task force report [3]. Value Heal. 2018;21:131–139. 10.1016/j.jval.2017.12.007 29477390

[9] Shafrin J, Kim J, Cohen JT, et al. Valuing the societal impact of medicines and other health technologies: A user guide to current best practices. Forum Heal Econ Policy. 2024;27:29–116. 10.1515/fhep-2024-0014 PMC11567015 39512185

[10] Neumann PJ, Garrison LP, Willke RJ. The history and future of the “ISPOR value flower”: Addressing limitations of conventional cost-effectiveness analysis. Value Heal. 2022;25:558–565. 10.1016/j.jval.2022.01.010 35279370

[11] Hogervorst MA, Møllebæk M, Vreman RA, et al. Perspectives on how to build bridges between regulation, health technology assessment and clinical guideline development: A qualitative focus group study with European experts. BMJ Open. 2023;13:e072309. 10.1136/bmjopen-2023-072309 PMC10462958 37640462

